# Dark septate endophytes isolated from a xerophyte plant promote the growth of *Ammopiptanthus mongolicus* under drought condition

**DOI:** 10.1038/s41598-018-26183-0

**Published:** 2018-05-21

**Authors:** Xia Li, Xueli He, Lifeng Hou, Ying Ren, Shaojie Wang, Fang Su

**Affiliations:** College of Life Sciences, Hebei University, Baoding, 071002 China

## Abstract

Dark septate endophytes (DSE) may facilitate plant growth and stress tolerance in stressful ecosystems. However, little is known about the response of plants to non-host DSE fungi isolated from other plants, especially under drought condition. This study aimed to seek and apply non-host DSE to evaluate their growth promoting effects in a desert species, *Ammopiptanthus mongolicus*, under drought condition. Nine DSE strains isolated from a super-xerophytic shrub, *Gymnocarpos przewalskii*, were identified and used as the non-host DSE. And DSE colonization rate (30–35%) and species composition in the roots of *G*. *przewalskii* were first reported. The inoculation results showed that all DSE strains were effective colonizers and formed a strain-dependent symbiosis with *A*. *mongolicus*. Specifically, one *Darksidea* strain, *Knufia* sp., and *Leptosphaeria* sp. increased the total biomass of *A*. *mongolicus* compared to non-inoculated plants. Two *Paraconiothyrium* strains, *Phialophora* sp., and *Embellisia chlamydospora* exhibited significantly positive effects on plant branch number, potassium and calcium content. Two *Paraconiothyrium* and *Darksidea* strains particularly decreased plant biomass or element content. As *A*. *mongolicus* plays important roles in fixing moving sand and delay desertification, the ability of certain DSE strains to promote desert plant growth indicates their potential use for vegetation recovery in arid environments.

## Introduction

Drought and desertification in China, especially in the northwest regions, are rapidly increasing and have become serious environmental problems limiting plant growth and revegetation^1,2^. While plants may exhibit improved drought resistance directly by means of altered morphology and physiology^3^, indirect plant responses to water stress via fungal symbionts have received less attention^4^. Using beneficial microbes to promote the establishment and growth of host plants within arid habitats has been considered a practical and useful bioprospecting strategy^4,5^. Thus, choosing fungi that were able to confer beneficial effects to plants appeared to be necessary.

As important root endophytes, arbuscular mycorrhizal fungi (AMF) and rhizobium are well documented and have significantly positive ecological roles in host plants^6,7^. However, dark septate endophytes (DSE), which are normally present in healthy plant roots, have only recently received more attention^8,9^. Fungi characterized by melanised, septate hyphae and microsclerotia-like structures in the roots of many plant species have been defined as DSE^10,11^, and they have been observed in the roots of approximately 600 plant species belonging to 110 families and 320 genera^11^. A meta-analysis studied the effects of DSE on host plants showed that DSE associations vary from negative to neutral and positive when measured by host performance or host tissue nutrient concentrations^12^. Similar to AMF, DSE exhibit positive effects on plant growth, water and nutrient uptake and increase the stress tolerance of host plants^9,13–18^.

DSE exhibit a broad distribution and high root colonization in a wide range of terrestrial ecosystems^8,9,14,19–23^, especially in extreme environments, including arid and semiarid environments^19,24–26^. To date, pure-culture and culture-independent molecular analyses indicated that many plant species in areas prone to drought stress harbour a variety of DSE species^19,24,26–28^. For example, Barrow^19^ surveyed the DSE colonization of *Bouteloua* sp. in arid southwestern USA rangelands and found DSE hyphae and microsclerotia within plant roots. Knapp *et al*.^25^ investigated DSE fungi of three invasive and eight native plants in semiarid sandy grasslands and showed that DSE fungi were frequently observed and represented approximately 60% of the isolates. Moreover, the main DSE groups showed no specificity in the colonization of native and invasive species. Northwest China has a vast landmass and rich species diversity, and the desert regions have numerous plant species. In our previous investigations in northwest China, DSE fungi were also found to co-occur with multiple desert plants, especially in extreme arid environments. For example, melanised septate hyphae and microsclerotia have been observed in the roots of *Ammopiptanthus mongolicus* and *Hedysarum scoparium*^29,30^. Typical DSE fungi, such as *Exophiala* sp., *Phialophora* sp. and *Phialocephala* sp. were isolated and identified from the roots of *A*. *mongolicus*^29^.

Fungi with melanised cell walls have been studied, and they showed increased resistance to heat and drought stress^31–33^. Thus, DSE fungi with melanised hyphae might play important roles in arid ecosystems. Studies using crops showed that DSE inoculation could increase the tolerance of rice plants to water deficiency^17^. In addition, DSE could also exhibited positive effects on non-host plants. For instance, DSE fungi isolated from maize could act be an effective colonizer, and improved the growth and drought resistance of sorghum seedlings^34^. For desert plants, although the distribution and abundance of DSE in (semi)arid regions have been widely investigated, studies of the association between DSE and desert plants under water stress are limited. To date, only one report has directly analysed the association between DSE and desert plants under drought condition. Perez-Naranjo^35^ conducted an inoculation experiment to study the influence of five DSE isolates on grass growth under water stress. The results showed that inoculation with DSE isolates stimulated the growth of *Agropyron cristatum* and *Psathyrostachys juncea* and inhibited the growth of *Bouteloua gracillis* under water deficiency.

Desert plants play critical roles in maintaining the sustainability of desert ecosystems^36^. *A*. *mongolicus* is the only evergreen broadleaf shrub, mainly distributed in the desert areas of northwest China^37^. This species has typical super xerophytic structures, and is particularly well suited for vegetation recovery as well as fixing moving sands and delaying further desertification^38^. During our previous studies, DSE fungi were observed in the roots of *A*. *mongolicus* and exhibit positive effects on host plants^29,39^. In order to seek and apply more beneficial DSE – *A*. *mongolicus* symbionts to combat desertification, we may wonder whether *A*. *mongolicus* can benefit from DSE fungi isolated from the other plants. *Gymnocarpos przewalskii*, with the growth of environmental and ecological distribution similar to that of *A*. *mongolicus*, is a super-xerophytic shrub restricted to extreme arid deserts of northwest China^40^. During the previous investigations on the AMF colonization status of *G*. *przewalskii*, DSE fungi were occasionally observed in the roots. Therefore, DSE resources of *G*. *przewalskii* were identified and selected for the inoculation experiment.

In this work, the main objectives were to determine whether non-host DSE fungi from extreme arid habitats could successfully colonize *A*. *mongolicus* seedlings and enhance plant growth under drought condition. Therefore, we first investigated and isolated DSE fungi in the roots of *G*. *przewalskii*. Second, an inoculation experiment was conducted to evaluate the growth of *A*. *mongolicus* response to these non-host DSE fungi under drought condition. We expect to answer the following questions: (1) What is the colonization and species composition of DSE fungi in the roots of *G*. *przewalskii* in the desert areas of northwest China? (2) Does these DSE fungi can act as non-host colonizer and affect the growth and nutrient uptake of *A*. *mongolicus* under artificial culture conditions? (3) What is the relationship between DSE fungi of *G*. *przewalskii* and *A*. *mongolicus*?

## Results

### Morphology of the DSE fungi in the roots of *Gymnocarpos przewalskii*

**Figure 1:**
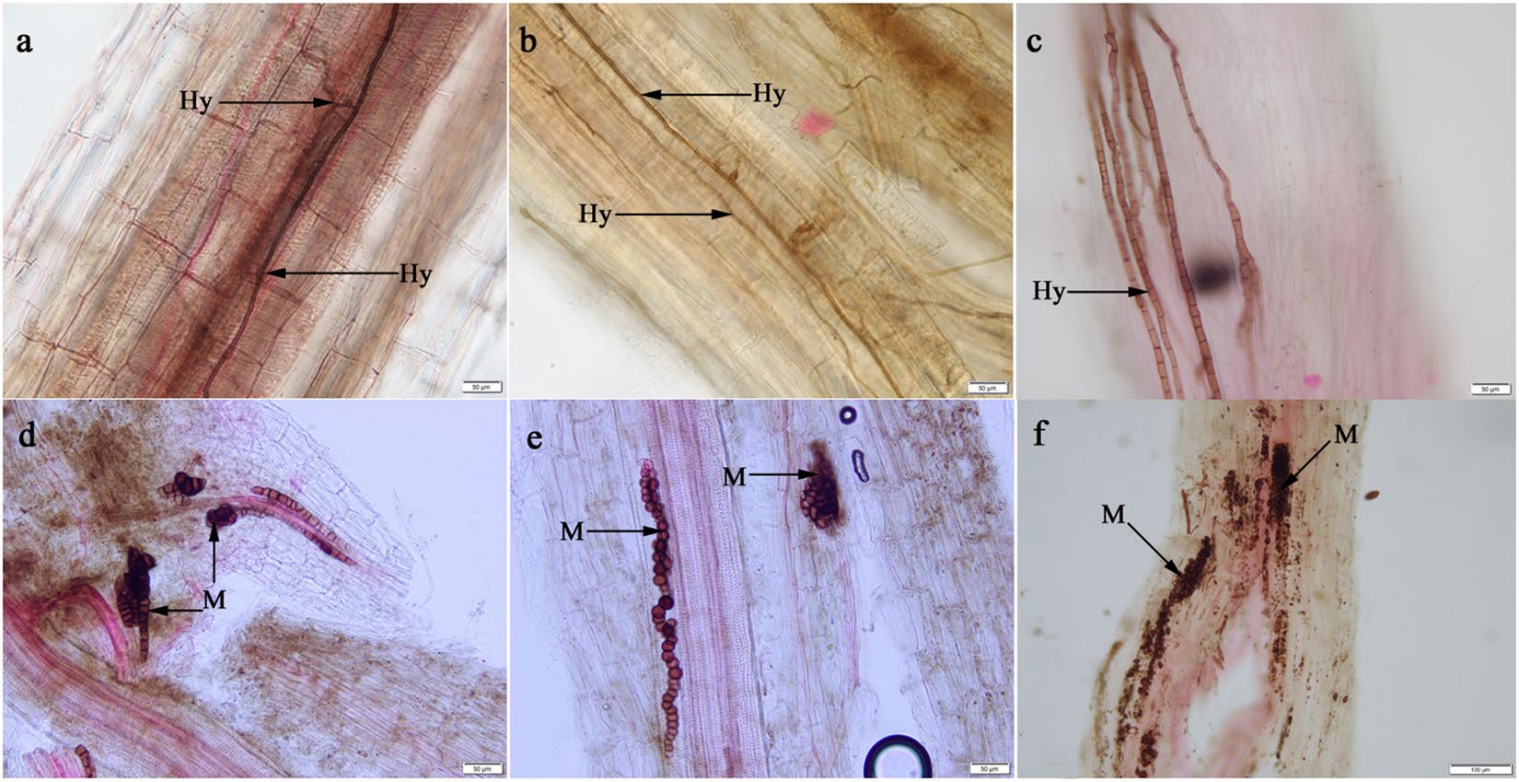
Dark septate endophytes associated with the roots of *Gymnocarpos przewalskii*. (**a**–**c**) DSE hyphae; (**d**–**f**) DSE microsclerotia. (**a**–**e**) bars = 50 µm; (**f**) bar = 100 µm. Arrows indicate the following: Hy, DSE hyphae; M, DSE microsclerotia.

Typical DSE hyphae and microsclerotia structures were observed in the roots of *G*. *przewalskii* (Fig. 1). Brown to dark brown septate hyphae with thick lateral walls invaded the epidermal or cortical cells (Fig. 1a–c). Chainlike and conglomerated microsclerotia filled single cortical cells or colonized more than one cell (Fig. 1d–f). DSE hyphae, microsclerotia and the total colonization rate in the surveyed plants of Minqin were approximately 30.7%, 6.7% and 30.0%, respectively, and the parameters for the surveyed plants of Anxi were approximately 18.0%, 22.0% and 35.0%, respectively.

**Figure 2:**
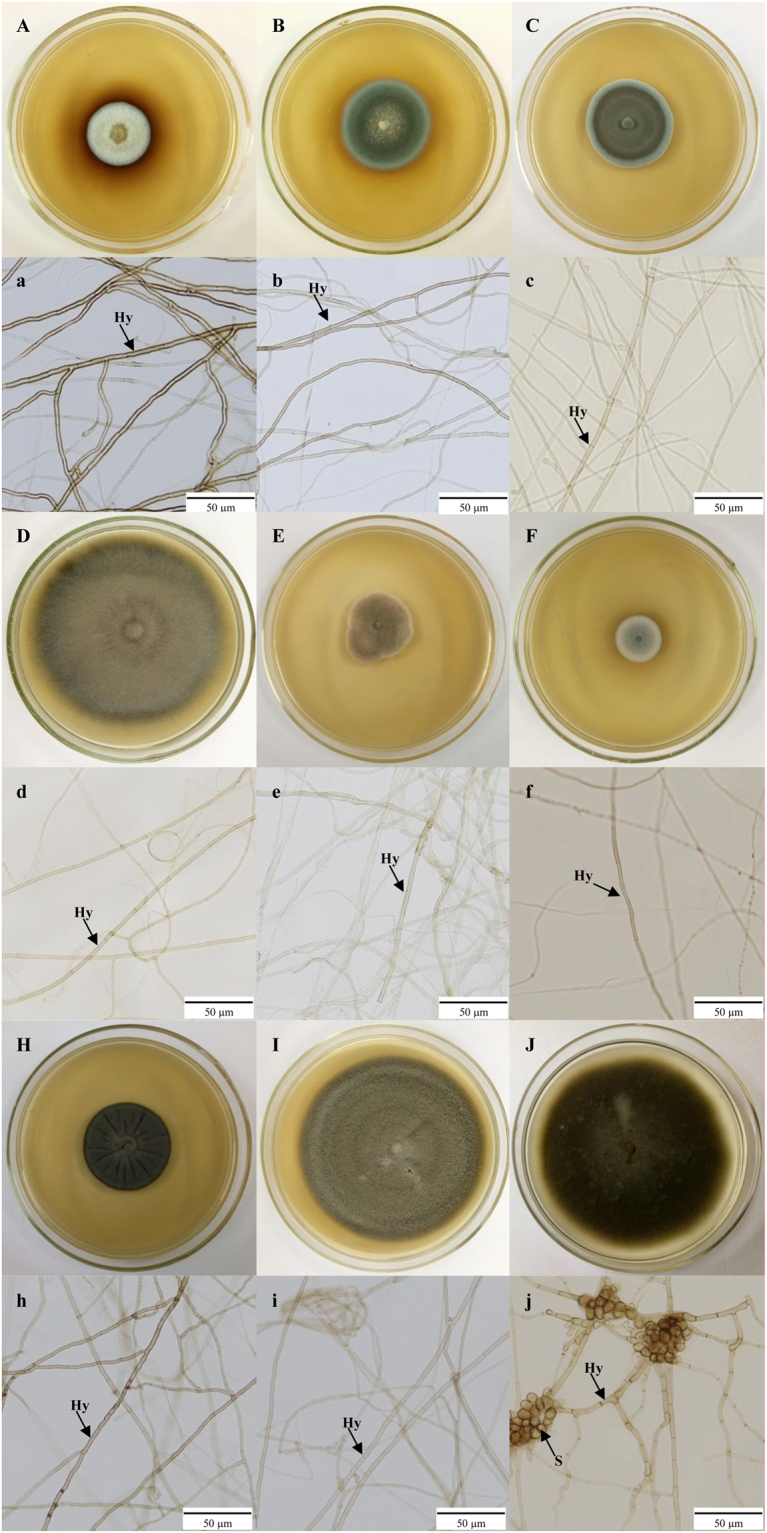
(**A**–**J**) Colonies of endophytic fungi isolated from roots of *Gymnocarpos przewalskii* on PDA medium. (**a**–**j**) Microscopic morphology of endophytic fungi (bars = 50 µm). (**A, a**) – isolate DSE 2; (**B, b**) – isolate DSE 3; (**C, c**) – isolate DSE 4; (**D, d**) – isolate DSE 6; (**E, e**) – isolate DSE 11; (**F, f**) – isolate DSE 13; (**H, h**) – isolate DSE 25; (**I, i**) – isolate DSE 27; and (**J, j**) – isolate DSE 28. Arrows indicate the following: Hy, DSE hyphae; S, DSE spores.

These nine DSE colonies isolated from *G*. *przewalskii* were ashen and grey to dark brown, and they are illustrated in Fig. 2. The growth curves of these colonies were linear, with average growth rates of 0.14, 0.21, 0.22, 0.48, 0.18, 0.21, 0.10, 0.19, 0.47, 0.86 cm/day. DSE 28 produced spores (Fig. 2j), whereas conidia or reproductive structures were not observed in the other isolates.

### Molecular phylogeny

**Figure 3:**
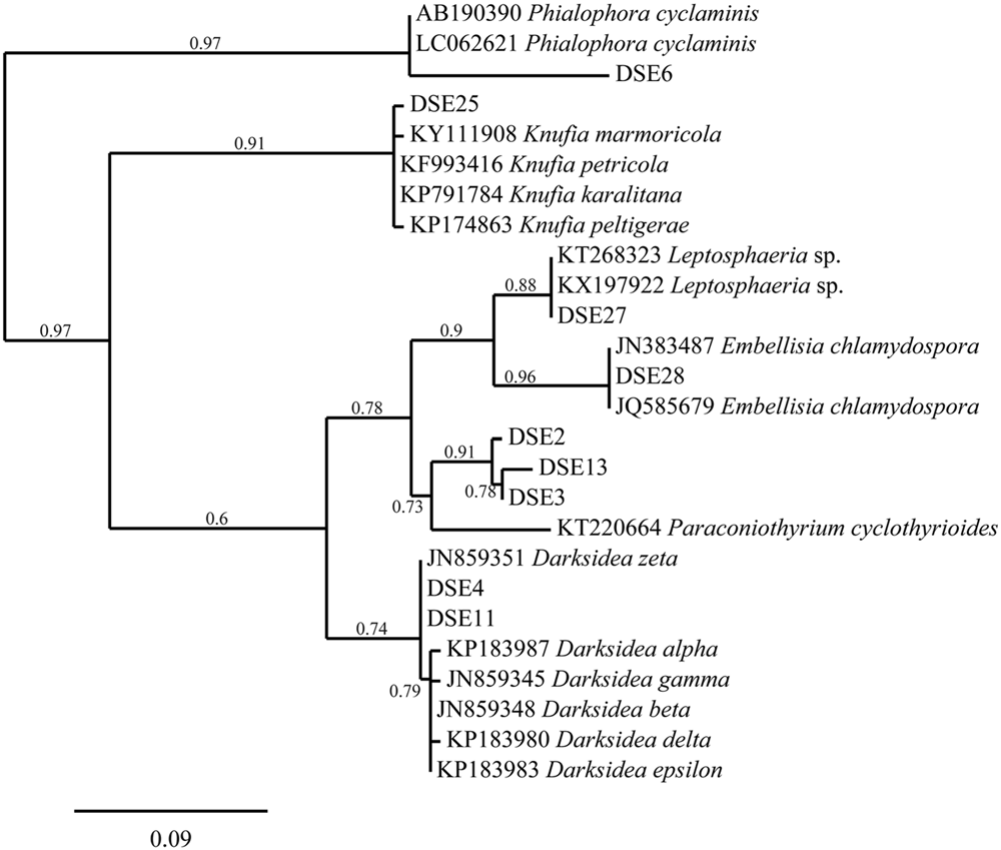
Maximum parsimony tree generated from ITS (ITS4 and ITS5) sequences of isolated strains and their closest matches followed by the GenBank accession number.

The phylogenesis of the ML tree based on the ITS4-5.8S-ITS5 rDNA is shown in Fig. 3. These isolates were clustered in six sequence groups. Based on the molecular phylogenetic analysis and morphological characteristics, the groups were identified as *Darksidea* sp., *Phialophora* sp., *Knufia* sp., *Leptosphaeria* sp. and *Embellisia chlamydospora* as well as three undescribed species of *Paraconiothyrium*. Among these isolates, Pleosporales was the dominant genera.

### Effect of DSE isolates on the growth of *Ammopiptanthus mongolicus* seedlings

**Figure 4:**
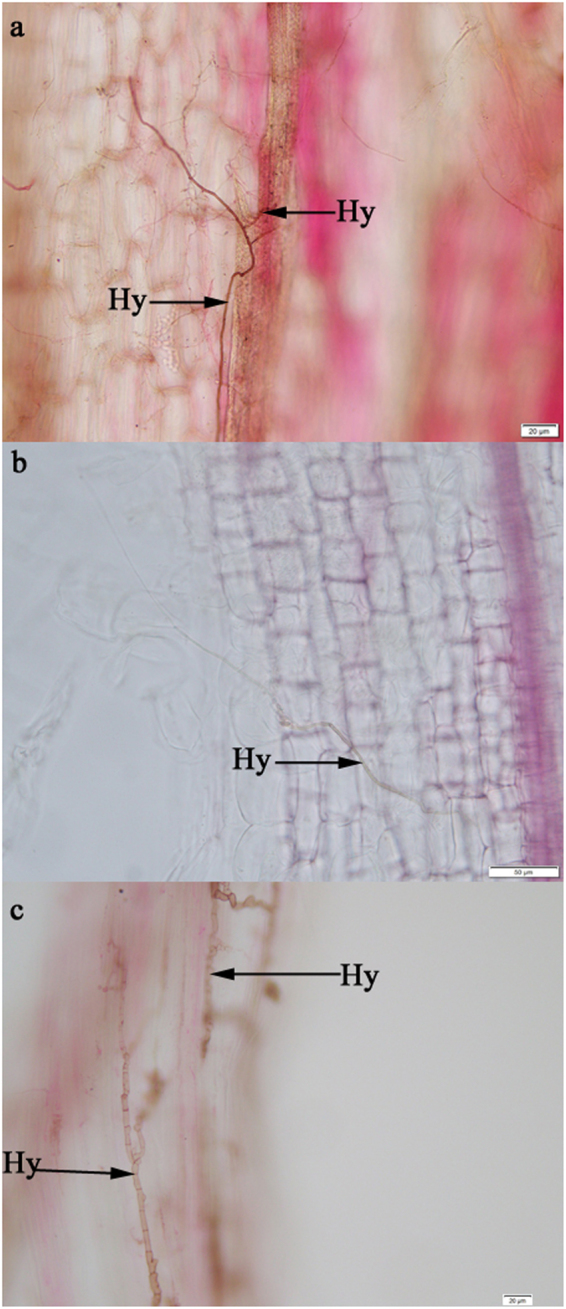
Colonization of certain DSE strains in the roots of inoculated *Ammopiptanthus mongolicus* seedlings after three months. Hy indicates DSE hyphae. (**a**) *Knufia* sp.; (**b**) *Phialophora* sp.; (**c**) *Embellisia chlamydospora*.

After three months of growth, all *A*. *mongolicus* seedlings were harvested except those inoculated with DSE 13. The leaves and stems of *A*. *mongolicus* inoculated with DSE 13 became chlorotic, and the plants were died at the end of the 90-d inoculation period. In contrast, the other colonized seedlings were green, alive and healthy. Microscopic observations showed that the roots of all *A*. *mongolicus* plants were colonized by DSE fungi isolated from *G*. *przewalskii* (Fig. 4).

DSE inoculation exhibited significant influences on the growth of *A*. *mongolicus*, and the effect varied with the DSE strain (Table 1). *Leptosphaeria* sp. (DSE 27) inoculation significantly increased the leaf number of *A*. *mongolicus* (68.3%) compared to the control treatment. All DSE isolates showed a positive effect on the branch number of *A*. *mongolicus*, except for *E. chlamydospora* (DSE 28), and *Paraconiothyrium* sp. (DSE 2, DSE 3) inoculated plants showed significantly higher values (increased by 110% and 120%) than non-inoculated plants. Inoculation with DSE did not significantly influence the plant height of the hosts, except for DSE 3, which showed a significant decrease by 33.0% compared to non-inoculated plants.

**Table 1:** Effects of DSE inoculation on the vegetative growth of *Ammopiptanthus mongolicus* plants.

Inoculation treatment	Leaf number	Branch number	Plant height	Root length
CK^a^	13.9 ± 1.42 bc	1.0 ± 0.00 c	5.4 ± 0.64 ab	4.0 ± 0.38 c
DSE2	21.3 ± 2.01 ab	2.1 ± 0.28 ab	5.2 ± 0.57 ab	4.9 ± 0.56 bc
DSE3	14.8 ± 2.64 bc	2.2 ± 0.25 a	3.6 ± 0.26 c	4.5 ± 0.37 bc
DSE4	19.9 ± 2.61 ab	1.4 ± 0.22 abc	5.4 ± 0.41 ab	6.3 ± 0.43 a
DSE6	16.9 ± 2.79 ab	1.3 ± 0.21 bc	5.9 ± 0.53 a	4.6 ± 0.29 bc
DSE11	9.0 ± 1.40 c	1.6 ± 0.27 abc	4.2 ± 0.19 bc	3.7 ± 0.30 c
DSE25	18.7 ± 3.03 ab	1.8 ± 0.36 abc	5.8 ± 0.34 a	6.7 ± 0.64 a
DSE27	23.4 ± 3.16 a	1.7 ± 0.42 abc	5.3 ± 0.43 ab	7.1 ± 0.63 a
DSE28	19.9 ± 1.23 ab	1.0 ± 0.00 c	6.0 ± 0.68 a	5.8 ± 0.49 ab

^a^CK indicates non-inoculated plants. Different letters indicate significant differences at *P* < 0.05.

### Effect of the DSE isolates on the element contents of *Ammopiptanthus mongolicus* seedlings

**Figure 5:**
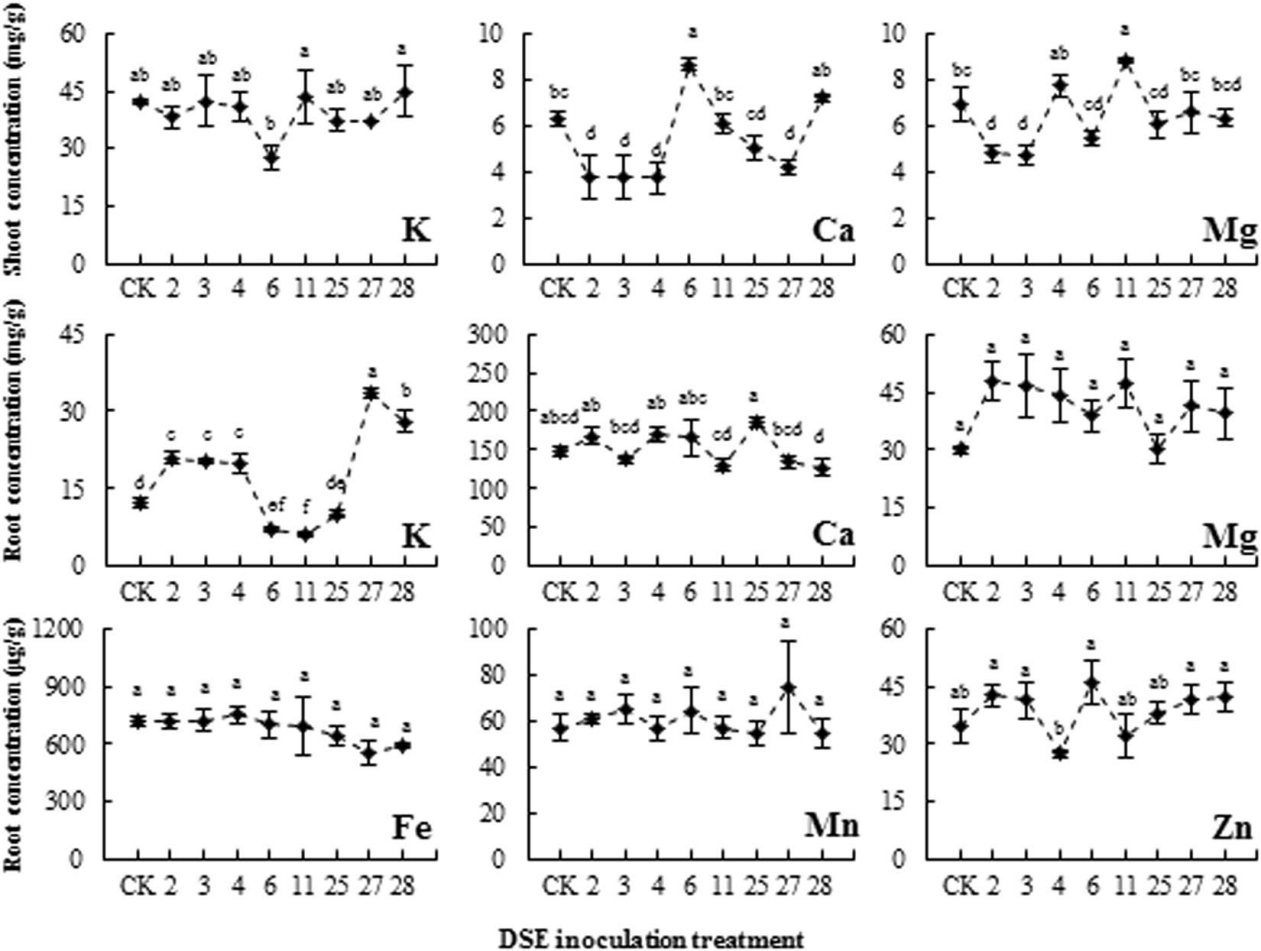
Effects of DSE inoculation on the element concentration of *Ammopiptanthus mongolicus*. CK indicates non-inoculated plants. Different letters indicate significant differences at *P* < 0.05.

Inoculation with *Paraconiothyrium* sp. (DSE 2, DSE 3), *Darksidea* sp. (DSE4), *Leptosphaeria* sp. (DSE27) and *E. chlamydospora* (DSE 28) resulted in a significant increase in the K content in the roots of *A*. *mongolicus* by 71.7%, 66.0%, 61.8%, 175.1%, and 130.0%, respectively, compared to CK plants. *Phialophora* sp. (DSE 6) induced a significant increase in the Ca content (36.8%) in the shoots, but a decrease in the K content (43.9%) in the roots. Compared with the control treatment, plants inoculated with DSE fungi did not show significant differences in the K content in the shoots and the Ca and Mg contents in the roots (Fig. 5). Plants inoculated with *Paraconiothyrium* sp. (DSE 2, DSE 3) and *Darksidea* sp. (DSE 4) exhibited decreased Ca content in the shoots, being about 60%, that of non-inoculated plants.

Microelements (Fe, Mn, Zn) were not detected in the shoots of inoculated and non-inoculated plants at the end of the experiment (Fig. 5), whereas in the roots, none of the inoculated *A*. *mongolicus* showed significant differences compared with the control (Fig. 5).

### Effect of DSE isolates on the biomass production of *Ammopiptanthus mongolicus* seedlings

**Figure 6:**
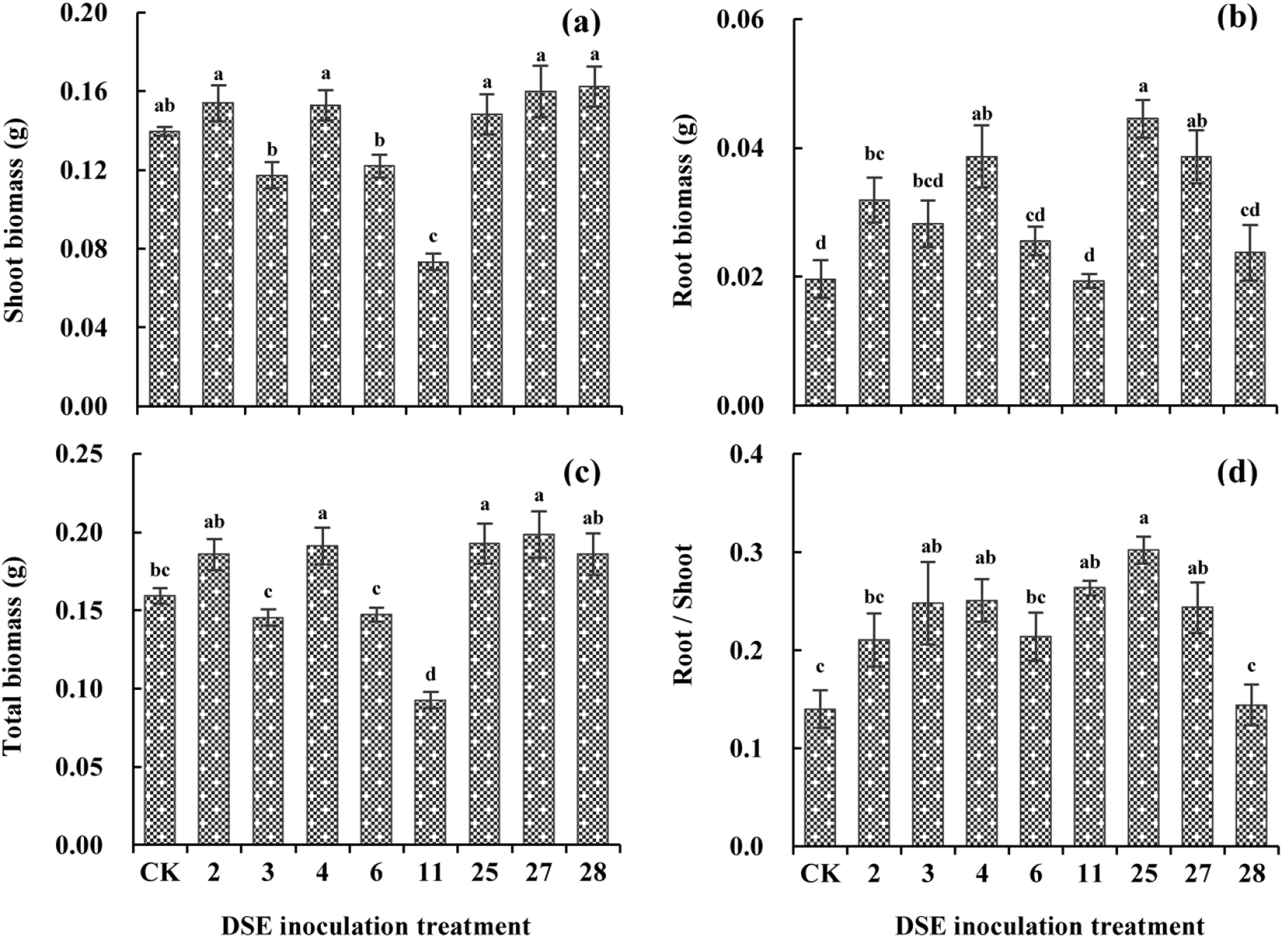
Effects of DSE inoculation on the biomass production of *Ammopiptanthus mongolicus*. CK indicates non-inoculated plants. Different letters indicate significant differences at *P* < 0.05.

DSE inoculation showed diverse effects on the biomass production of *A*. *mongolicus*, and the effects varied with the DSE strain (Fig. 6). All DSE isolates, except for DSE 11, increased the root biomass of *A*. *mongolicus* compared to CK plants, and one *Paraconiothyrium* strain (DSE 2), *Darksidea* sp. (DSE 4), *Knufia* sp. (DSE 25), and *Leptosphaeria* sp. (DSE 27) were significantly higher for this variable (increased by 62.5%, 96.8%, 126.9%, and 96.4%) (Fig. 6b). The total biomass of *Darksidea* sp. (DSE 4), *Knufia* sp. (DSE 25), and *Leptosphaeria* sp. (DSE 27) inoculated plants were significantly higher (20.2%, 21.1%, and 24.7%) than those of non-inoculated plants (Fig. 6c). One *Paraconiothyrium* strain (DSE 3), *Darksidea* sp. (DSE 4), *Knufia* sp. (DSE 25), and *Leptosphaeria* sp. (DSE 27) were observed a significant promote effect on the root/shoot ratio of *A*. *mongolicus* (77.4%, 79.3%, 115.8%, and 74.0%) compared with that of the non-inoculated plants, whereas the other DSE isolates showed negative or neutral influences (Fig. 6d). *Darksidea* sp. (DSE 11) displayed a significant negative effect on the shoot biomass (47.5%) and total biomass (41.9%) of *A*. *mongolicus* compared with that of non-inoculated plants (Fig. 6). Among the remainder of the DSE isolates, inoculation did not have a significant influence on the shoot biomass of the plants (Fig. 6a).

## Discussion

### Investigation and identification of DSE in the roots of *Gymnocarpos przewalskii*

Field investigations revealed that DSE are distributed worldwide as root-endophytic fungi and represent frequent colonists of plants growing under extreme conditions^8,14,19,20,22,24,25^. In this study, typical melanized DSE mycelium and microsclerotia in the roots of *G*. *przewalskii* were observed in more than half of all sample plants distributed in a desert sandy area of northwest China. This study provides the first record of DSE associations in *G*. *przewalskii*. For these two sample sites, DSE exhibited different survival mode that were likely related to the degree of soil drought. Compared with the plants at the Anxi site, the roots of *G*. *przewalskii* collected from Minqin showed higher DSE hyphal colonization and lower DSE microsclerotia colonization. Microsclerotia are speculated to serve as an important storage substances and may play a role in nutrient uptake and (or) accumulation^41^. For example, *Asparagus officinalis* colonized by the dark septate fungus *Phialocephala fortinii* were studied by Yu *et al*.^41^, and the results showed that microsclerotia accumulated high levels of glycogen, protein, polyphosphate, and phosphorus. Thus, we speculate that the presence of additional microsclerotia in plant roots in the Anxi site, where the water stress condition was worse, might act as a protective device against drought stress and more microsclerotia may means greater ability to withstand arid conditions.

Many results suggest that DSE fungi are generalists and colonize several hosts^11,25^. Similarly, the DSE isolates in this study were grouped with several endophytes from different geographic and host origins. For example, *Knufia* sp. and *Leptosphaeria* sp. were previously observed in *Populus* sp. and coastal grasses^42,43^. *Darksidea* sp., *Phialophora* sp., and *E. chlamydospora* have been reported in certain desert plants from semiarid areas on the Great Hungarian Plain and in China^25,28,30^. In addition, DSE 6, which belongs to *Phialophora* sp., was reported in *A*. *mongolicus* in our previous publication^29^. Both DSE 6 and the other DSE successfully colonized the roots of *A*. *mongolicus* at the end of the inoculation experiment. Our results supported previous conclusions that DSE were non-host-specific^20,25^, indicating that DSE have the potential for widespread use in host plants other than the original host.

### Effects of DSE inoculation on the growth of *Ammopiptanthus mongolicus*

The broad distribution of DSE in drought-prone habitats suggests that they might have important functions for plant survival in desert ecosystems^19,24,25,28^. However, information on the functions of DSE in desert ecosystems is still insufficient. To date, the positive effects of DSE on host performance under drought stress have been reported for certain grasses. Perez-Naranjo^35^ found that five but one DSE isolates resulted in a positive growth response in *A*. *cristatum* or *P*. *juncea* under water stress; however, none of the DSE isolates provided benefits to *B*. *gracillis*. Santos *et al*.^17^ also studied the effects of DSE inoculation on the performance of a wild rice species (*Oryza glumaepatula*) under water stress conditions and found that DSE increased the tolerance of rice plants to drought stress. In this study, all DSE strains were able to act as non-host colonizers, and formed a selectively symbiosis with *A*. *mongolicus* that was dependent on the DSE species. Inoculation with different DSE species exhibited positive (DSE 4, DSE 25, and DSE 27), neutral (DSE 2, DSE 3, DSE 6, and DSE 28), and negative (DSE 11, DSE13) effects on the growth of *A*. *mongolicus*, such as total biomass. This finding is consistent with the reports of Wilcox and Wang^44^, who inoculated four DSE fungi in three species of trees and found that certain DSE fungi were either weak or serious pathogens, whereas others appeared to promote host growth. Previous studies showed inconsistent results on the effects of DSE on the biomass of host plants^9,45,46^. Our results indicated that DSE fungal species may be one of the factors that govern whether the symbiotic relationship is mutualistic^12,35,44,47^.

Although certain DSE isolates had neutral effects on the total biomass accumulation of inoculated plants, they exhibited positive influences on other growth parameters. Reports have indicated that DSE can show pathogenic or mutualistic effects on hosts depending on the growth conditions^48,49^. Verbruggen and Kiers^50^ also argued that positive rather than negative interactions between DSE and hosts were more likely to emerge and persist in stressful environments. In the present study, the soil water content was slightly higher (10%) than that in natural habitats (1–7%). The positive influence of these DSE fungi may play a more important role in plant growth under lower water supply conditions.

### Potential mechanisms for the mutualistic behaviour of DSE isolates

Two possible reasons can explain the enhanced growth of *A*. *mongolicus* inoculated with DSE. First, DSE increased the nutrient uptake of hosts. Barrow^19^ proposed that DSE fungi formed a fungal network associated with a mucilaginous complex on the host root surface that might enhance nutrient transport in roots exposed to very low water potentials. Other studies have indicated that the facilitation of nutrient uptake (nitrogen or phosphorus contents) in the tissues of hosts was an important factor^9,11,12,51^. In the present study, we further observed that DSE inoculation (DSE 4 and DSE 27) promoted the acquisition of K in the roots of *A*. *mongolicus*. Similar results regarding shoot K content were observed in Polar plants colonized by *Leptodontidium* sp.^52^ K is an indispensable component required by plants, and it might also play an important role in the osmotic adjustment of *A*. *mongolicus* under water-stressed conditions as previously reported^53^. Second, the production of phytohormones by DSE fungi^54–56^ or their indirect regulation affects the hormone production of host plants^57–59^. For example, Rudawska *et al*.^60^ found that DSE fungi were able to release indole acetic acid. Although the production of phytohormones was not measured in this study, *A*. *mongolicus* inoculated with DSE4, DSE25, and DSE27 exhibited a longer root length and higher root biomass compared with that of the non-inoculated plants. We suspect that the beneficial effect of DSE on host roots might also be related to increased hormone production. However, the detailed mechanism by which DSE promotes the growth of its host still requires further research.

### Potential application of DSE fungi in deserts

With the climate changes and extensive human activities, desertification has becoming more and more serious^38^. Thus, improved ability of the sand-fixing plant – *A*. *mongolicus* to overcome drought stress is important for the sustainable conservation in deserts. In this study, the main positive effect of DSE inoculation on *A*. *mongolicus* was enhanced root biomass and branch growth, which might facilitate its adaptation to arid environments. With decreasing annual precipitation in arid regions (<100–200 mm), ground evaporation intensifies and acts primarily near the ground surface^61^. The enhanced root growth (such as root biomass and root/shoot ratio) can facilitate the extension of *A*. *mongolicus* roots to deeper soil layers and improve water extraction from the soil, which allows the plant to avoid ground evaporation and promotes its vigorous survival in desert ecosystems^62^. Moreover, wind erosion is one of the principal drivers of land deterioration in arid environments^63^. When desert ecosystems suffer serious desertification, their surfaces become sensitized to wind erosion and sand drifts^64,65^. The presence of additional root branches and stems can slow winds and stabilize sand, thus facilitating the deposition of nearby sand and seeds^66,67^. This effect is also beneficial for the subsequent establishment of more plants^68,69^. In conclusion, the ability of certain non-host DSE strains to promote root and branch growth of *A*. *mongolicus* might facilitate the vegetative recovery in desert ecosystems.

## Conclusion

In conclusion, DSE colonization and species composition in the roots of *G*. *przewalskii* distributed in the deserts of northwest China were first reported. DSE fungi from *G*. *przewalskii* could improve the growth of *A*. *mongolicus* under drought stress, but this beneficial effect was dependent on DSE species. Subsequent research should further investigate the effect of DSE on plants under more extreme drought treatment.

## Methods

### Study sites and sampling

Root samples of *G*. *przewalskii* were collected at two sites: the Anxi Extra-Arid Desert National Nature Reserve (N38°05′, E103°02′) and the Minqin Liangucheng National Nature Reserve (N40°52′, E95°78′), Gansu Province, northwest China. These areas have a typical arid continental climate with remarkable seasonal and diurnal temperature variations. The average annual precipitation is 45.7 mm and 113.9 mm in Anxi and Minqin, respectively. The soils at each site are characterized by desert sands, and the vegetation is dominated by *G*. *przewalskii*. Soil moisture was determined by soil humidity recorder (L99-TWS-2, China). The volumetric water content percentage of the sample soil in Anxi and Minqin is approximately 1–4% and 3–7%, respectively. The physicochemical characteristics of the soils in Anxi and Minqin were as follows: pH 7.65 and 7.47, organic matter 1.2 and 1.8 g/kg, alkali-hydro N 14.26 and 8.12 mg/kg, available P 1.2 and 1.8 mg/kg, respectively.

Three sample patches were randomly selected at each site at a distance of ≥100 m. Fine root samples in the rhizosphere of five *G*. *przewalskii* were collected from each site in July 2015. The totally 30 root samples were collected at a depth of 30 cm in each patch, placed in sealed plastic bags and transported to the laboratory in an insulated container within 48 h.

### Microscopic analysis

Fresh roots were cleaned using tap water to remove soil particles and placed into 10% (w/v) potassium hydroxide for 1 h at 100 °C. The cleaned roots were stained with 0.5% (w/v) acid fuchsin^70^ for 20 min at 90 °C. For each plant, approximately 20 randomly selected 0.5-cm-long segments were examined microscopically at 200× and 400× magnification^71^. The colonization rate of DSE hyphae, microsclerotial, and total (%) were analysed as follows:

Colonization rate (%) = (length of colonized root segments/total length of root segments) × 100%.

### Isolation of endophytic fungi

Approximately 5 randomly selected 0.5-cm-long root segments from each plant were selected for the isolation of DSE. The roots were surface sterilized by dipping them in 70% ethanol for 5 min and then 5% sodium hypochlorite for 5 min under agitation and then washing three times in sterile distilled water. These roots were then transferred to potato dextrose agar (PDA) culture medium supplemented with antibiotics (ampicillin and streptomycin sulphate) and kept at 27 °C in the dark. To make sure the isolates are true endophytes, 200 µL of the distilled water left in the final step were coated on PDA midium as a contrast. Mycelium growing from the cut ends of root segments were transferred to new PDA plates and kept in the dark at 27 °C before performing the macroscopic observations and measurements^72^. Totally, 37 colonies were isolated from 150 root segments. Isolates showed similar morphology and growth rate were grouped into 9 morphotypes. Therefore, 9 typical isolates (DSE2, DSE3, DSE4, DSE6, DSE11, DSE13, DSE25, DSE27, and DSE28) were selected for the following research (Fig. 2). The colony diameter was measured every two days for 14 d. The measurement was performed for at least 5 replicates per isolate. Moreover, each isolate had three replicates that were cultured at 10 °C for two months to induce sporogenesis^28^.

### Molecular identification of endophytic fungi

Fresh mycelia (approximately 50 mg) were scraped from the surface of PDA plates, and DNA was extracted using a genomic DNA extraction kit (Solarbio, China). Two primers, ITS4 (5′–TCC TCC GCT TAT TGA TAT GC–3′) and ITS5 (5′–GGA AGT AAA AGT CGT AAC AAG G–3′), were used for all isolates. PCR was performed in 40 µL volumes containing 7 µL of fungal genomic DNA, 1 µL of each primer, 20 µL of 2× Es Taq Master Mix, and 11 µL of ddH_2_O. PCR cycling was performed in a Life ECO^TM^ system (BIOER, China) using the following programme: initial denaturation at 94 °C for 5 min; followed by 35 cycles of 94 °C 1 min, 55 °C 1 min, 72 °C 1 min; and a final incubation at 72 °C for 10 min^30^. The PCR products were purified and sequenced, and then sequences were deposited in GenBank under the accession numbers MF035998-MF036006. The sequences were aligned via MUSCLE with G-block curation^73^, and the tree was inferred by the maximum likelihood (ML) method via PhyML^74^ implemented at the phylogeny.fr website^75^. Branch-support values of the phylogenetic tree were estimated using the approximate likelihood-ratio test^76^ with the SH-like option.

### Preparation of DSE-inoculated and non-inoculated *Ammopiptanthus mongolicus* seedlings

For this experiment, *A*. *mongolicus* seedlings were inoculated with all 9 isolates or sterile PDA disks. *A*. *mongolicus* seeds were surface sterilized by dipping into 70% ethanol for 3 min and 2.5% sodium hypochlorite for 10 min and then rinsing three times in sterile distilled water. The sterilized seeds were aseptically placed on the water agar medium (agar 10 g/L) for germination at 27 °C. Sand collected from the Anxi and Minqin sites were mixed and sieved through a 0.2-cm sieve and then autoclaved for 90 min at 121 °C. The mixed sand contained 0.21% organic matter, 11 mg/kg available nitrogen and 1.5 mg/kg available phosphorus. One-week-old seedlings were transplanted into sterile glass bottles (Φ 60 × 120 mm) containing culture substrata for fungal inoculation. All inoculation processes were performed on a super clean bench. Each glass bottle was filled with 270 g sterile culture substrate containing 250 g sand mixed with 20 mL Murashige and Skoog (MS) liquid medium. Approximately 10% soil water content was chosen for the drought stress treatment to avoid the death of *A*. *mongolicus* seedlings. Two germinating seedlings were planted in each sterile microcosm. Four fungal disks (Φ 0.5 cm) cut from a 14-days-old DSE culture were inoculated at a 1-cm range close to the roots of *A*. *mongolicus* seedlings^77^. The non-inoculated controls were inoculated with plugs excised from the PDA plate without fungus. In total, 10 plants were treated with each fungal isolate or sterile PDA plugs. The cultures were placed in a growth chamber at 27 °C (50% humidity) with a 12 h light/12 h dark photoperiod.

### Harvest of *Ammopiptanthus mongolicus* seedlings

Plant growth parameters, such as the leaf number, branch number, and plant height, were measured for each plant. The shoots (leaves and stems) and roots of each microcosm were carefully separated, and the roots were first assessed for DSE colonization status as described above. The shoots and roots were separately placed in a 70 °C oven for 48 h and then weighed. The dried shoot (0.1 g) and root samples (0.03 g) were ground separately and burned in a muffle furnace at 550 °C for at least 5 h, and then the residue was dissolved in nitric acid for sample digestion, totally three replicates. The major elements (Potassium, K; Calcium, Ca; and Magnesium, Mg) and microelements (Iron, Fe; Manganese, Mn; and Zine, Zn) were measured using the flame atom absorption spectrophotometer.

### Statistical analyses

All statistical analyses were performed with SPSS 21.0 (SPSS, Chicago). The differences in plant growth parameters (leaf number, branch number, height, and root length), plant biomass and element concentrations in the shoots and roots between the inoculated and non-inoculated plants were determined via a one-way analysis of variance (ANOVA). Differences between the means among different treatments were compared using Duncan’s multiple-range tests at probability levels of 0.05.
